# Intraoral Applications of Lasers in the Prosthetic Rehabilitation with Fixed Partial Dentures—A Narrative Review

**DOI:** 10.3390/dj12060164

**Published:** 2024-05-31

**Authors:** Magdalena Kwaśna, Paulina Cłapińska, Zuzanna Piosik, Kamila Barysz, Iga Dubiec, Adam Bęben, Iwona Ordyniec-Kwaśnica

**Affiliations:** Department of Dental Prosthetics, Medical University of Gdańsk, Elizy Orzeszkowej 18, 80-208 Gdańsk, Poland; paulina.clapinska@gumed.edu.pl (P.C.); zuza.ps@gumed.edu.pl (Z.P.); kbarysz@gumed.edu.pl (K.B.); idubiec@gumed.edu.pl (I.D.); adam.beben@gumed.edu.pl (A.B.); iwona.ordyniec-kwasnica@gumed.edu.pl (I.O.-K.)

**Keywords:** prosthodontics, Nd:YAG laser, Er:YAG laser, dentistry, diode laser, laser-assisted dentistry

## Abstract

Laser, an acronym for Light Amplification by Stimulated Emission of Radiation, is a powerful tool with diverse applications in modern dentistry. It emits monochromatic, coherent light resulting from photon-induced chain reactions. Available dental lasers include diode, argon, Er,Cr:YSGG, Er:YAG, Nd:YAG, and CO_2_. The unique property of these lasers, allowing them to be effectively used on both soft and hard tissues based on the operational parameters, positions them as particularly suited for a wide range of dental procedures. Compared to traditional methods, lasers offer advantages such as improved hemostasis and quicker wound healing. Such benefits stress the shift towards laser technology in dental treatment. In the realm of dental prosthodontics, which focuses on esthetics, functionality, and the physiological aspects of dental prostheses, lasers provide promising outcomes. Among the prosthetic options, fixed partial dentures stand out for their ability to mimic natural teeth, offering both esthetic and functional features, leading to satisfactory long-term outcomes if managed properly. This review paper delves into the specific application of laser technology in the context of prosthetic rehabilitation involving fixed partial dentures. By investigating intraoral laser procedures, it contributes to understanding laser’s role in improving patients’ satisfaction and clinical efficiency in this field.

## 1. Introduction

In 1917, Albert Einstein laid the groundwork for laser technology by introducing the theory of stimulated emission, which underpins the scientific principles behind the creation of laser light [1]. The first use of lasers in dental practice dates back to 1956, when Thomas Maiman created the prototype of the ruby laser (694 nm). He subjected an extracted tooth to his model tool, which led to energy transfer [1,2]. The laser, which is an acronym for Light Amplification by Stimulated Emission of Radiation, represents a versatile tool with multiple applications in modern dentistry [2,3]. It emits monochromatic and coherent light, which originates from the release of additional photons that initiate a series of chain reactions [3].

The peak development of laser dentistry occurred in the mid-1990s [1,2], providing dentists with lasers such as diode (655–980 nm), Nd:YAG (1064 nm), Er,Cr:YSGG (2780 nm), Er:YAG (2940 nm), and CO_2_ (10,600 nm) [1,2,4]. It is observable that dental lasers cover the spectra of ultraviolet, visible light, and infrared.

Lasers can be classified according to the laser active material, laser movement, level of energy emission, or range of wavelength [5]. The wavelength produced by the laser determines its mechanism of action. Depending on this parameter, the wave is absorbed by substances such as water, hemoglobin, hydroxyapatite, and melanin, which allows the physician to obtain a therapeutic effect in the target tissue [1,6]. Laser classifications and their parameters are summed up in Table 1. Table 2 provides a detailed comparison of the benefits and drawbacks associated with each dental laser. This table systematically lists the pros and cons to help to understand their impact in dental practices [4,5,7]. It serves as a valuable resource for evaluating the effectiveness of different laser types in dentistry.

Lasers technology represents a contemporary and appealing tool, offering a multitude of advantages over conventional methods. Its low invasiveness, immediate hemostasis, and faster wound healing attract practitioners in many fields of dentistry [1]. Lasers have been found to have the potential to control bacteriemia [8] and reduce the occurrence of medical emergencies among medically compromised patients [9], as well as those suffering from hemorrhagic disorders [10,11,12,13,14]. Despite these beneficial characteristics, it is noted that lasers are not used to their full potential and there is a lack of complete understanding of their benefits among dental professionals [15].

The rapid technological advancement of laser therapy, coupled with the constant expansion of its applications in treating hard and soft tissues, encourages the search for its novel clinical applications within dental prosthodontics. The simultaneous development of esthetic dentistry along with the increasing needs of patients compels prosthodontists to adopt a holistic approach to therapy. In aging populations, tooth wear becomes increasingly significant, which is driven by attrition (tooth-to-tooth wear), erosion (acid-induced enamel loss), and abrasion (wear from external objects like toothbrushes). These conditions can escalate due to longer lifespans, dietary acids, medications, and health issues like acid reflux, leading to heightened tooth sensitivity, decay, and structural damage [16,17]. The holistic approach not only addresses the restoration or replacement of teeth but also factors in the overall oral ecosystem and the patient’s psychological well-being, ensuring a more successful and satisfying result within the limits of functional and physiological capabilities [18,19].

Fixed partial dentures appear to be favored by patients due to their ease of use, natural tooth-like appearance, and satisfactory long-term results if designed and assembled properly [20,21]. Different materials for fixed prosthetics can be selected based on specific needs, but it is crucial that they are appropriate for the individual case and offer optimal tribological properties [17,22].

**Figure 1:**
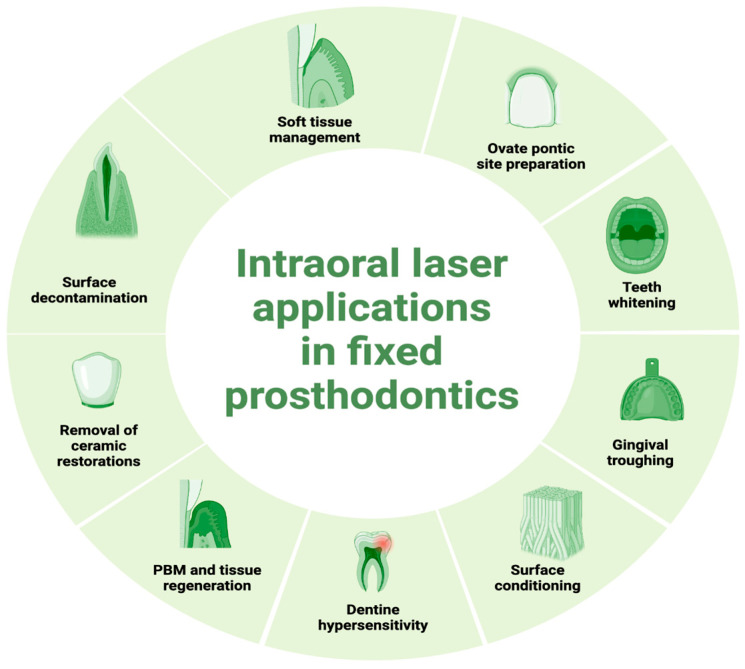
Presentation of laser applications in prosthetic treatment in fixed prosthodontics.

The article emphasizes how laser technology enhances dental procedures and highlights its potential in improving outcomes in prosthetic rehabilitation in relation to fixed partial dentures. Its aim is to expand the understanding and usage of lasers in dental prosthetics, advocating for further research and integration into clinical practice to leverage its benefits fully. The applications of lasers detailed in this article are presented in Figure 1.

## 2. Intraoral Applications of Lasers

### 2.1. Soft Tissue Management

Lasers can be applied in prosthodontic procedures such as crown lengthening and soft tissue management. The latter specifically refers to the removal and reconstruction of gingival tissue around abutments and laminates like veneers. The prime examples of gingival reshaping procedures are gingivectomy and gingivoplasty. They enhance esthetic appearance, especially for individuals with a type of smile characterized by excessive gum visibility, referred to as a gummy smile. Conventional methods for managing this ailment include injections with botulinum toxin into the muscle group responsible for upper lip lifting. However, laser gingivectomy offers better results. Studies were conducted comparing the effectiveness of the traditional method with a procedure where a diode laser in continuous mode was used [23]. The group of patients that received the botulinum toxin injection experienced the improvement a week after the procedure and the effects lasted for a limited time, around 4–6 months. Oppositely, the second group treated with laser noticed the gummy smiles recession immediately after the procedure and did not require any corrections after several months.

The conventional tools used to lengthen clinical crowns consist of scalpels and rotary instruments. A comparison study was conducted to assess the effectiveness of the common approach and lasers. Patients were divided into two groups. The surgery on the first group was conducted using the standard tools, while the second group was treated with a 2780 nm wavelength Er,Cr:YSGG laser [24]. The specific parameters and the mode used were not provided. Immediately after the surgery, a significant increase in the length of the clinical crown was observed in both groups. However, after 3 months, the first group experienced a recession of the gum margin and a regression to the original condition. In opposition, the second group retained the margin at an almost-constant, previously determined level. Additionally, the sealing of blood vessels was observed using the laser method, contributing to fewer mechanical injuries, reduced bleeding, and lesser swelling. It is worth noting that the laser surgery also lasts for a shorter time and contributes to limiting the post-treatment discomfort. It also enables easier continuity of the further stages of treatment such as taking intraoral impressions.

**Figure 2:**
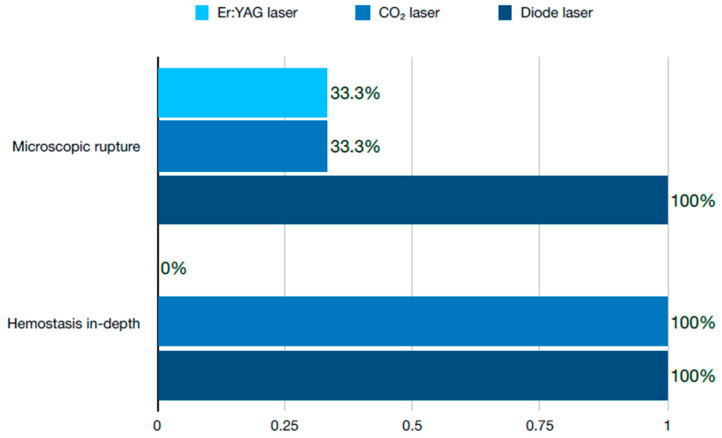
Microscopic rupture of the tissue and hemostasis in-depth depending on the laser used.

**Figure 3:**
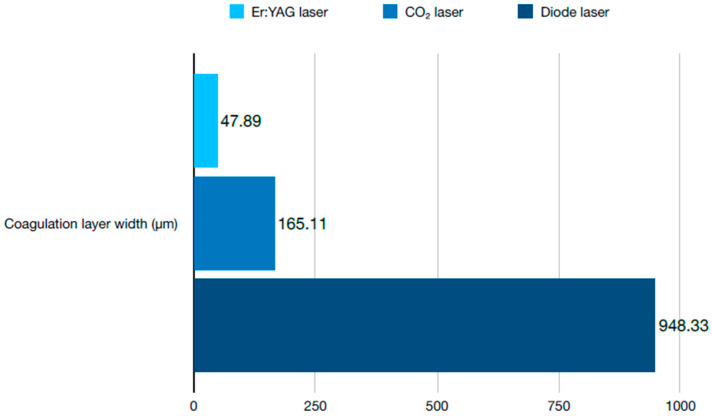
Coagulation layer width in μm depending on the laser used.

To trace the effect of different procedures on the oral tissue, a study was carried out that compared the areas of the gum incision after gingivectomy with tools such as Er:YAG, CO_2_, and diode lasers with scalpel cuts [25]. The first two of the modern devices were set to pulsed mode, while the latter was set to continuous mode. Histological parameters such as the thickness of the coagulation layer, the presence or absence of microscopic rupture, and hemostasis were measured for all the sections of the tissue. The most promising results were obtained with the CO_2_ laser which demonstrated high hemostasis, minimal microscopic ruptures, and a thin coagulation layer. The aforementioned features of the lasers display a significant advantage to the surgical scalpel, as they contribute to a faster healing process, reduced pain, and postoperative swelling. A comparison of the results with the above lasers is presented in Figure 2 and Figure 3.

Gingival plastic laser procedures provide excellent clinical results, but at the same time they require detailed and specialized knowledge. An in vitro study was conducted to determine the most appropriate irradiation time and laser power to avoid potentially harmful temperature increases in the pulp of the tooth [26]. A total of 90 freshly extracted teeth—30 front teeth, 30 premolars, and 30 molars—were utilized. The study concluded that the use of a diode laser in continuous mode, at 1 W in the anterior, premolar, and molar teeth for 20, 40, and 60 s, respectively, generates an acceptable increase in pulp temperature. In comparison, the power of 2 W used for the same irradiation time showed a potentially harmful effect. Additionally, it was noted that the dark discoloration occurring on the surfaces of the teeth has a negative impact on the temperature fluctuation in the pulp. This observation proves that the scaling and cleaning of tooth surfaces before carrying out the planned procedures should provide beneficial effects.

### 2.2. Preparation of Ovate Pontic Site

Replacing missing teeth in the anterior region demands particular attention due to the extended esthetic, phonetic, and hygienic requirements. Concurrently, those cannot compromise the function of the dental prosthesis used [20].

In accordance with literary sources, all these features are met by ovate pontic, if using a prosthetic bridge as the chosen restoration [20,27]. It provides exceptional esthetic results due to a similar emergence profile as the natural tooth [20]. Additionally, ovate pontic supports and maintains the interdental papillae, which subsequently eliminates the presence of black triangles [27]. However, this pontic design necessitates a sufficient amount of hard and soft tissues that need to be shaped appropriately [20].

There are a number of conventional methods used to provide a desired recipient site for the ovate pontic. These are based on using long-term provisional restorations directly after tooth extraction or involve gingivoplasty, which is performed with high-speed rotary instruments or electrosurgery [27].

**Figure 4:**
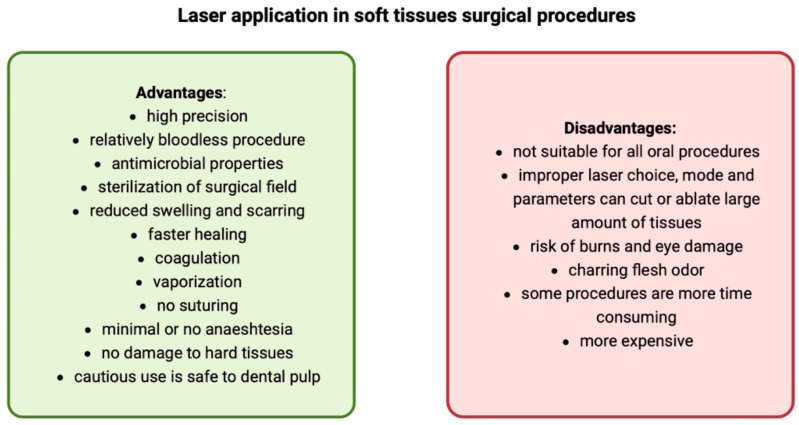
Advantages and disadvantages of laser application in soft tissues surgical procedures.

Currently, the ovate pontic site can be obtained using a diode laser [28,29]. There are a number of benefits with using this method in comparison to the traditional approach, including low invasiveness, providing a sterile surgical field, hemostasis, precise modulation of the soft tissues, reduction in postoperative pain and swelling, faster wound healing, and a shorter operation time [29,30]. The laser technology also allows the procedure to be conducted in the initial treatment session, contrary to the conventional methods which require execution several weeks prior to the preparation/impression stage [29]. The advantages and disadvantages of laser-assisted surgical procedures in soft tissues are presented in Figure 4 [30].

Research on laser-assisted ovate pontic site preparation is limited, resulting in the absence of a standardized protocol. The existing studies employed a 980 nm diode laser in continuous-wave contact mode with a 2.5 W fiber tip [28], and an 810 nm diode laser in gated pulse-wave mode with an average power of 1.5 W [29]. Despite the differences in approach, both methods report positive postoperative outcomes and emphasize satisfying esthetic results of the final restoration alongside overall patient comfort during the procedure.

### 2.3. Tooth Whitening

**Figure 5:**
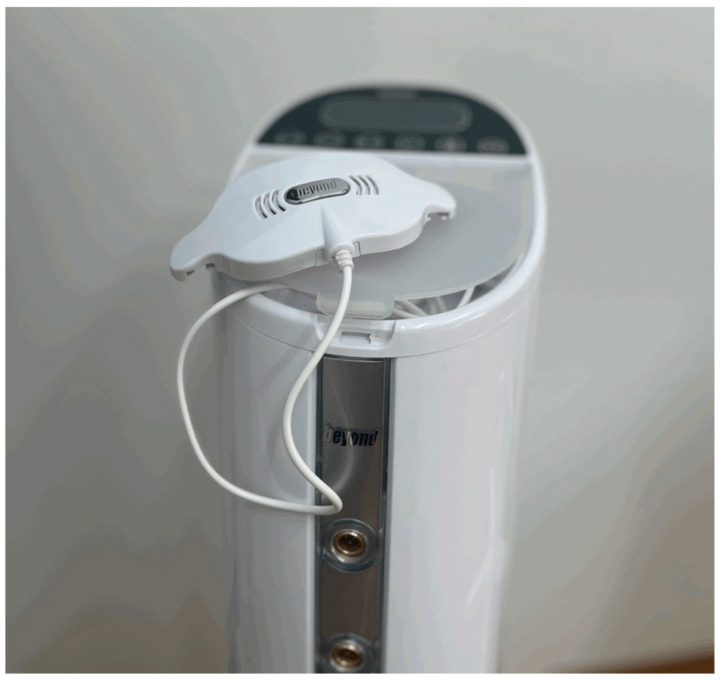
A lamp for laser teeth whitening with a wavelength of 480–520 nm.

One of the most common treatments to improve your smile is tooth whitening. Although this is not a necessary condition for the use of prosthetics, in some cases this treatment can complement and optimize prosthetic therapy [31,32]. Tooth whitening can be performed professionally in a dental office during a visit or as a home treatment, commonly known as overlay whitening. The method used by dentists involves applying a whitening gel to the surface of the patient’s teeth, which is then activated by the light of a special lamp or continuous-wave laser. An example of such a lamp is shown in the Figure 5.

One clinical case in which tooth whitening before prosthetic reconstruction may be very beneficial is the treatment of teeth discolored with tetracyclines. This preparatory step often leads to more esthetically pleasing results when veneers or crowns are later placed. It allows a minimal reduction in tooth structure because the desired whiteness is achieved earlier, reducing the need to use thicker or more opaque materials to mask discoloration. Moreover, whitening effects can reach deeper layers of the tooth, ensuring accurate and stable staining, providing a consistent base for prosthetics [33].

There are many factors that can affect the effectiveness of the whitening process, such as oral hygiene, exposure time to the bleaching agent, the concentration of hydrogen peroxide in the bleaching agent, or the energy source used to initiate and accelerate the chemical reaction [32]. Therefore, it is impossible to establish a single, universal tooth whitening protocol that would be the most effective and applicable in every case. Additionally, most studies have limitations due to the fact that only immediate whitening results were assessed, which does not provide information on the long-term stability of whitening. The tests are carried out on teeth in vitro, where there is no risk of the overheating of tooth tissues, which must be taken into account when whitening teeth in vivo.

In laser tooth whitening, the laser beam is responsible for accelerating the process of releasing free radicals from the whitening gel, which significantly shortens the duration of the procedure [31]. Lasers used for teeth whitening include argon, CO_2_, and diode lasers [34]. It has been shown that all of the above-mentioned lasers are characterized by a higher effectiveness in lightening tooth color compared to mechanical or light-activated lamp whitening [32].

An important difference between lasers used for whitening is the texture of the tooth structure after treatment [35]. It has been shown that diode lasers have the least impact on changing surface roughness and are the most effective in maintaining the integrity of the enamel. These features make the diode laser a laser used primarily in dental practice for whitening purposes [36].

In the wavelength spectrum of a diode laser, longer wavelengths are better absorbed by the whitening gel than shorter wavelengths. However, this leads to an increase in temperature and greater molecular mobility of the gel, which results in a greater release and penetration of hydrogen peroxide into the pulp chamber [37].

### 2.4. Gingival Troughing

Designing a fixed partial denture requires proper tooth preparation followed by taking an impression. Surgical troughing with a laser can offer greater benefits than other surgical and non-surgical methods [38].

While all preparations require gingival retraction, the subgingival placement is where tissue management is especially important. There is a need for the subgingival placement of the preparation in cases of extensive caries, previous restorations, crown fractures, retention, and esthetic demand. Subgingival margin preparation requires accuracy during impression-taking to ensure a perfect copy of the hard tissues of the tooth and the finishing line. The elastomeric impression materials are known to have hydrophobic properties and their use requires the absolute dryness of the gingival sulcus and retraction of the free gingiva by at least 0.2 mm [39]. It is worth remembering that no subsequent stage of preparing the restoration will be able to compensate for inadequate impression at this point; therefore, the success of the treatment depends on this stage [38].

Gingival retraction can be achieved by using mechanical or chemomechanical techniques or surgical techniques called troughing, based on the removal of the sulcular epithelium with rotary porcelain tools and high-energy lasers: diode, Er:YAG, and Nd:YAG. [40,41]. Table 3 below, created by Tao et al. [41], shows the laser operation parameters used for soft tissue management.

The use of laser troughing as an alternative to chemomechanical methods has been proven to provide the same necessary conditions to obtain a correct impression. It is also shown that lasers can reduce the negative aspects of the gingival retraction risk of postoperative complications, thus contributing to the success of prosthetic treatment [42]. It has been tested that all of the above-mentioned lasers achieved the necessary values of tissue displacement, which proves they are a viable option of gum retraction [43].

To investigate the effect of laser retraction on gum recession, the height of the gingival margin was compared, on the day of retraction and up to 8 weeks after surgery. The Er:YAG laser achieved lower recession values than the single impregnated cord [40,41]. The diode laser achieved a lower degree of recession when used on the anterior teeth compared to the posterior section. When molars and premolars are considered, there is no evidence of the superiority of the diode laser over the chemomechanical retraction technique [43]. It is possible for this effect to be related to the gum biotype, thickness of the gum, and tissue color [40].

The main advantages of laser troughing compared to gingival retraction are the simultaneous tissue biomodulation and precisely controlled procedure. It results in pain reduction during the procedure, immediate hemostasis, and a shorter treatment time, which benefits the patient’s overall comfort during the visit. Moreover, it leads to reduced inflammation and tissue swelling and no damage to the epithelial attachment and the basal layer of the epithelium is made. In addition, the use of a laser may offer better retraction and healing conditions for people with coagulation defects or those who have a lower regenerative capacity due to comorbidities [40].

### 2.5. Surface Conditioning

The preparation of the tooth and the surface conditioning performed prior to the cementation of the fixed prosthetic has a significant impact on the contact tightness and durability of prosthetic restoration. For this purpose, research has been carried out to find the most effective method for conditioning the surface of enamel and dentin to test the bond strength between the tooth, the cement, and the restoration material.

In order to apply adhesive techniques in clinical practice, it is necessary to etch the enamel layer. Until recently, only the classic method by Buonocore using hydrofluoric acid was used. This technique is based on the selective dissolution of the enamel to achieve microporosity and thus mechanical retention [44]. A more recent procedure involves the use of a laser on the enamel layer to melt and re-crystallize hydroxyapatite, which results in the roughening of the surface to a similar microporosity as the acid etching creates. The main advantage of laser surface conditioning is that it is less likely to damage the enamel. However, the subject literature also describes some disadvantages of lasers, i.e., the undesirable thermal side effects and the formation of micro-cracks. The review conducted by Labunet et al. [44] investigated the effectiveness of four types of lasers on the enamel layer: Er:YAG, Er,Cr:YSGG, Nd:YAG, and CO_2_. The lasers were tested according to their wavelength spectrum, power, mode of operation (pulsed, continuous), and exposure time. The results demonstrated that the most effective method was etching the enamel with the Er:YAG laser as it is less aggressive on the hard tissues and at the same time very efficient in achieving the desired microretention pattern. The Er,Cr:YSGG laser provided slightly worse outcomes compared to Er:YAG. Like the previous one, it increased the surface roughness and removed the smear layer without causing cracks. However, its use was associated with a higher risk of damage to the enamel as a result of thermo-mechanical ablation, a mechanism that causes micro-cracks. Lastly, the use of Nd:YAG and CO_2_ lasers on hard tissue has not surpassed the conventional acid etching.

Lithium disilicate ceramic material is gaining popularity as the prosthetic restoration material of choice due to its cosmetic value and high bending resistance. The etching procedure for conditioning the restorations made out of lithium disilicate traditionally uses hydrofluoric acid (HF) to create microretention on the surface. However, researchers have discovered that high-energy lasers such as Er:YAG and Nd:YAG lasers used in combination with the HF acid etch can increase the bonding strength of the resin-ceramic [45].

Another extremely valuable ceramic material is zirconium oxide, used to make durable and highly esthetic permanent restorations. However, achieving compatibility with cement remains a significant challenge. The standard procedure uses sandblasting to create micro-roughness on the surface, but to mitigate its negative effects, alternative lasers such as Nd:YAG, Er:YAG, and Er,Cr:YSGG, and continuous-wave carbon dioxide (CO_2_) have been used. However, Er:YAG and CO_2_ lasers can cause micro-cracks in the surface, which may result in a lower flexural strength.

As the research shows, the most effective laser for processing zirconium oxide surfaces is the femtosecond laser, which is not one of the lasers used in dentistry. The main advantages of this laser are that it increases the roughness of zirconium oxide and the content of the monoclinic phase without causing thermal damage [46]. As confirmed in many studies, it produces ultra-short, high-intensity pulses, very high frequencies, and very high scanning speeds [47]. In addition, laser microstructuring created very predictable, uniform, highly repeatable, parallel surface grooves on dental ceramics without a loss of bending strength and increasing the Weibull modulus and, consequently, the reliability of the material [48].

Therefore, lasers provide better retention on the surface of permanent dental restorations due to the different physical process of micro-roughness compared to chemical etching. These methods can reduce microcracks leading to microleakage and the lower durability of the cemented restoration.

### 2.6. Surface Decontamination

The bond strength between the tooth surface and restoration is affected by the presence of saliva, blood, and the smear layer; therefore, disinfection and decontamination procedures are a necessary step in surface preparation [49]. Photodynamic therapy and low-energy lasers can remove the microbial contamination from the hard tissues from the tooth and the restoration surface and lead to a better retention of fixed prostheses, as well as a lower risk of microleakage and bacterial recolonization [50].

PDT performed using a diode laser set to 660 nm in continuous pulse mode coupled with methylene blue and a diode laser set to 810 nm coupled with indocyanine green as photosensitizers showed the total eradication of *Streptococcus mutans* in an in vitro study by Azizi et al. [50].

Another study by Mishra et al. [51] assessed the specific operation procedure of a diode laser at 980 nm in two settings, 3 W continuous mode for 20 s, 3 W pulse mode 20 s in comparison to rinsing with 5.25% sodium hypochlorite for 5 min, and the continuous mode achieved the highest level of bacterial decontamination, followed by sodium hypochlorite and then the diode laser in pulse mode.

Root canal treatment can be finalized by the placement of a post cemented with adhesive resin. Success is guaranteed by a perfect seal and the removal of the smear layer and bacterial contamination. Air–water cooling can effectively remove the smear layer via ablation when combined with an Er,Cr:YSGG laser used at 2780 nm, in one of two modes: a 400 µm radial tip and 20 Hz frequency, or a 415 µm axial tip and 15 Hz frequency, both modes with an energy of 1.2 W. The debridement procedure can also be performed using Er:YAG, Nd:YAG lasers. In this study, the Er,Cr:YSGG laser has enhanced bonding strength with self-adhesive cement (RElyX Unicem Aplicap) and not with others tested; yet, in all groups, bacterial reduction has been achieved [52].

Additionally, diode lasers can be used to perform the decontamination of the surface of crowns placed on implants. In the operation mode at 810 nm, with a fiber tip 600 µm in diameter, in the manufacturer’s L-mode, a 25 W/PM:15,000 Hz/10 us/3.84 W pulses (Elexxion claros AG, Singen, Germany) diode laser has a biocidal effect without altering the structure of zirconia and porcelain restoration. This property is significant in preventing biofilm creation leading to periodontitis and peri-implantitis. The diode laser was operated in three modes of single, double, or triple irradiation of 15 s; while all modes reduced the amount of bacteria on the surface, the 45 s irradiation achieved a disinfection efficiency of over 90% in 7 out of 12 bacterial samples [53].

In conclusion, photodynamic procedures can be implemented in the prosthodontic treatment process before cementing fixed restoration to enhance the decontamination of the tooth structure and ensure a perfect seal, and also change the bonding conditions for adhesive cements. The disinfection procedure is part of the surface conditioning process when using a high-energy Er,Cr:YSGG laser, but it can also be performed with low-energy diode lasers without altering the surface structure [51,52,53].

### 2.7. Dentine Hypersensitivity

Dentine hypersensitivity is a multifactorial pathology defined as sharp pain caused by the exposure of dentinal tubules, in response to thermal, chemical, or mechanical stimuli [54,55,56]. It is one of the most common ailments reported by dental patients [54,57]. Its presence may cause an obstacle to prosthetic treatment, as well as a discomfort for patients, hence the need for its elimination. Traditional treatment consists of using desensitizing agents, for instance, fluorine or glutaraldehyde preparations [54]. However, since the 1980s, lasers have been demonstrating increasing significance [55,57].

**Figure 6:**
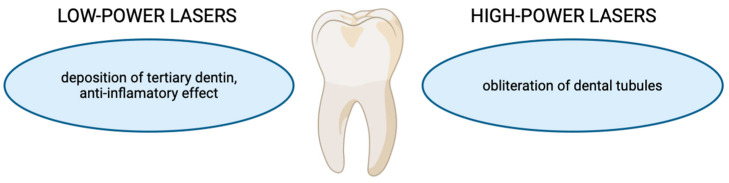
A summary presenting the effects of low-power and high-power lasers in the treatment of dentine hypersensitivity.

Many types of lasers are used to treat dentin hypersensitivity, including both low-power and high-power lasers. Among them, it is possible to distinguish the He-Ne, GaAlAs diode, Nd:YAG, Er:YAG, and CO_2_ lasers [55,57]. The mechanism of action of low-power lasers is associated with obliterating dentine tubules by stimulating the deposition of tertiary dentin [54]. It also has anti-inflammatory and analgesic effects [54]. On the other hand, high-power lasers cause obliteration of the dentin tubules through the fusion and then solidification of hydroxyapatite crystals [54,56]. These effects are summed up in Figure 6. Nevertheless, a single appropriate laser therapy protocol for dental hypersensitivity has not yet been established. Different types of lasers, wavelengths, potencies, and application times and modes have been used by researchers over the years [54,56].

One of the significant advantages of using lasers for the treatment of this complaint is related to their immediate effects [54,55]. Additively, they are also safe and well-tolerated by patients [54,55]. However, many researchers indicate a similar treatment effectiveness achieved when only topical desensitizing agents are used by patients [57], while others report benefits resulting from the simultaneous combination of these preparations and laser therapy [55]. Due to the comparable results of treatment with low- and high-power lasers, the low lasers, for example the diode lasers, appear to be a more affordable option for dental practitioners [54].

### 2.8. Photobiomodulation and Tissue Regeneration

Numerous studies indicate the connection between periodontal health and the longevity of the fixed partial dentures [58]. The poor condition of the periodontium can affect prosthetic treatment from the moment of the tooth preparation, through impression taking and the cementation process, up to the final esthetic outcome [59,60]. It is emphasized that there should be no signs of inflammation in either the soft or hard tissues during the luting process, nor at the end of the prosthetic rehabilitation [60].

Photobiomodulation (PBM), also known as low-level laser therapy (LLLT), involves the exposure of cells or tissues to low doses of red or near-infrared light [61]. The reaction of the tissue to the energy transferred during PBM exhibits a biphasic dose-response: too low of a dose may not demonstrate any therapeutic effect, the optimal dose can reveal biostimulatory properties, while excessive doses result in bioinhibition [62].

**Figure 7:**
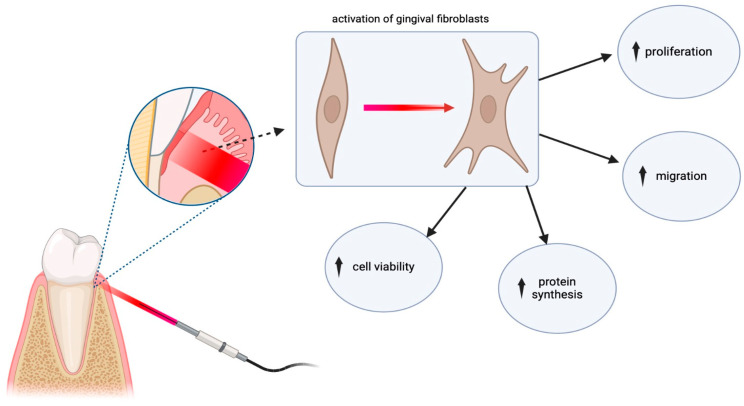
Presentation of the effect of PBM on gingival fibroblasts.

LLLT has gained recognition for its ability to stimulate cellular regeneration and expedite the wound healing process. The field of dentistry exhibiting particular interest in the use of low-level laser therapy is periodontology. There are a number of in vitro studies which have examined the effect of PBM at the cellular level on cell migration, viability, proliferation capacity, differentiation, protein synthesis, or growth factors’ gene expression, presenting the beneficial effects of this method on the activity of gingival fibroblasts [61,63]. The effects of PBM on gingival fibroblasts are presented in Figure 7.

Moreover, PBM shows satisfactory results in terms of reducing inflammation and alleviating pain [64]. Hence, it appears reasonable to assume that LLLT may prove to be a useful method, ensuring uninterrupted prosthetic treatment by eliminating the need to postpone visits caused by a poor periodontal tissue condition. Furthermore, PBM could lead to achieving a more appealing effect of the final fixed dental restoration. This is supported by case reports of patients who underwent prosthetic rehabilitation assisted by LLLT at every stage of the treatment. Positive outcomes were obtained in both the condition of the periodontium and the esthetic result of the final restoration [60,65]. Both reports detailed the use of a diode laser (660 nm) applied at specific points, but varied in the number of points, the energy delivered, and the number of sessions and intervals between them. Specifically, one report used the laser in four sessions spaced 2–3 days apart, at six points with 0.1 J per point [60]. The other report conducted six sessions with weekly intervals at two points applying an energy of 1 J per point [65].

Particular consideration should be made in terms of applying PBM during prosthetic treatment among elderly people. With age, the regenerative and defensive functions of the human body deteriorate, which can lead to chronic inflammation or impaired wound healing, including in the oral cavity [66]. The results of an in vitro study, which examined the biostimulatory effect of LLLT (780 nm, 4 min time exposure in a continuous wave, three radiations performed at 24 h intervals at 3.0 J/cm^2^ energy density) on gum tissue derived from young (18–25 years old) and old (over 65 years old) patients, reveal multiple beneficial effects of this therapy on cell viability, migration, and collagen and VEGF synthesis within gingival fibroblasts in both study groups [67]. Nevertheless, further examination, including in vivo studies, of PBM therapy within the older demographic is necessary to evaluate its effectiveness. Particular emphasis should be put on patients suffering from chronic illnesses affecting their regenerating abilities. 

A novel method within prosthetic dentistry that incorporates PBM is hemolasertherapy. It aims at diminishing the black triangles between the teeth, which are caused by atrophy of the interdental papilla or its anatomical distortion [68]. The research indicates that photobiomodulation of the gingival tissue and the stem cells contained in it results in the bioactivation of these cells, improved blood circulation, fibroblasts’ stimulation, and local metabolic improvement [68,69]. This in turn may lead to improvement in the size of the interdental papilla or even complete filling of the empty space, sometimes as early as 14 days after the procedure [68]. The study utilized a 660 nm diode laser, which was applied at seven points, with each point receiving 2 J of energy. This application occurred in two sessions, one week apart, and was conducted both before and after inducing bleeding [68].

This technique can positively affect final esthetic results, eliminating the need to compromise the shape of the dental restoration to conceal the interdental gaps.

### 2.9. Removal of Ceramic Restorations

Fixed dental restorations have gained importance in dentistry, especially in the restoration of damaged, cracked, or missing teeth. An array of ceramic materials is used by dental technicians to construct crowns or veneers. Some of them, for instance, lithium disilicate, ensure magnificent esthetics due to excellent optical effects and the ability to imitate enamel and dentin [70]. Other types of materials, such as zirconia, provide a high mechanical strength [70].

**Figure 8:**
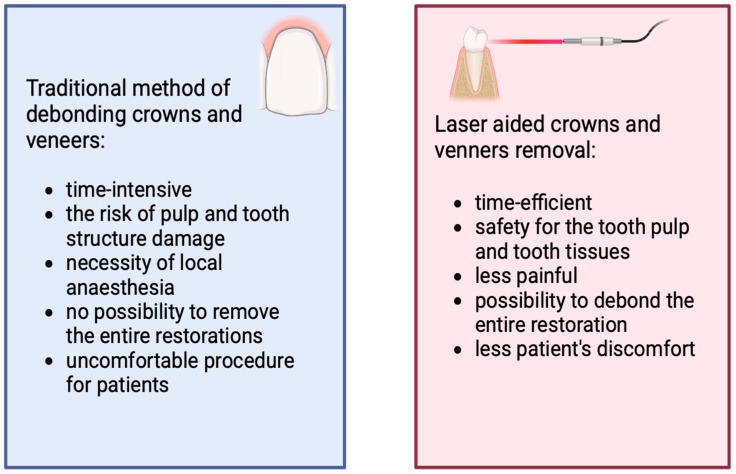
The comparison of traditional and laser approaches of fixed dental restorations removal.

The popularity of ceramic restorations in dental practice presents a challenge for dentists related to their removal for functional or esthetic failures [70]. The traditional method of debonding all-ceramic crowns or veneers involves using a diamond [71] or tungsten carbide burs [70,72] with appropriate air–water cooling. This approach is associated with numerous disadvantages, such as the difficulty of the procedure, the possibility of the pulp’s thermal damage, the necessity of using local anesthesia, and particular precision. This is because restorations, bonding cement, and the underlying dentin all share a similar color [70,71]. As a consequence, damage of the tooth structure is highly possible [73]. Furthermore, the conventional removal is time-consuming [70,71]. A comparison of the traditional and laser methods of debonding is presented in Figure 8. Due to the above, lasers are becoming more and more important tools in prosthetics. Two types are used to remove ceramic dental restorations: Er,Cr:YSGG (2780 nm) and Er:YAG (2940 nm) [71,73]. Both of these are applied in a pulsed mode [70,72], but additional clinical trials are required to establish an accurate protocol for the removal of fixed partial dentures.

Research indicates that laser energy is penetrated through the ceramic, contributing to the evaporation of the resin cements’ components like residual monomers in a mechanism called thermal ablation [70]. As a result, dentists can debond and remove entire ceramic restorations from teeth without producing iatrogenic damage of the enamel or dentin [73,74]. This, in turn, enables the correction or repair process outside a patient’s oral cavity and then recementing of the restorations [71]. The conventional method of using rotary instruments is always associated with damaging the integrity of the ceramic [73]. An essential advantage of using lasers is also the lack of the tooth structure damage, which could lead for instance to the patient’s postoperative hypersensitivity. The absence of modifications within the tooth structure, as well as removal of all the resin cement, was observed under a scanning electron microscope (SEM) [74]. An ex vivo study conducted by Zhang et al. confirmed that the residual tooth structure is not altered if a low laser fluency (19.94 J/cm^2^) is used [74]. More precisely, an Er:YAG laser with a sapphire tip was applied for veneer debonding, employing non-contact mode and air–water cooling. The laser’s pulse duration was set at 800 μs, with scanning performed both horizontally and vertically across the veneer surface. All these parameters constitute a guarantee of minimizing the risk of damage to the tooth tissues [74].

What is more, the application of lasers is linked to the working time’s reduction, which has a positive impact on the patient’s comfort. All-ceramic crowns made of lithium disilicate or zirconium oxide are effectively removed between 30 and 120 s, while thinner veneers, with a thickness of less than 1 mm, are effectively removed between 9 and 15 s [70]. Therefore, the working time depends on the thickness and type of ceramic used to make a fixed dental restoration. For comparison, the removal time of lithium disilicate crowns using diamond burs in the laboratory is, on average, 6 min [71].

Laser technology used by appropriately trained prosthodontics is also safe for tooth pulp. Studies carried out using an Er:YAG laser on 20 extracted molars covered with full-contour lithium disilicate crowns showed that the temperature increase in the pulp chamber was on average 5.4 ± 2.2 °C [72]. In contemporary dentistry, it is believed that an intrapulpal temperature rise of 5.5 °C can contribute to irreversible pulpal necrosis [70,72]. However, in the conducted study, an increase in the temperature inside the pulp above 5.5 °C was observed only when air–water cooling was applied on the side of the tooth that was opposite to the direction of irradiation [72]. Moreover, neither a constant heat increase nor heat accumulation was observed inside the pulp chamber [72]. Therefore, in order to avoid possible thermal damage to the pulp due to temperature spikes or insufficient heat diffusion, appropriate air–water cooling should be applied [72].

## 3. Limitations

The current research, framed as a narrative review, inherently carries several limitations due to its broad scope, which encompasses multiple applications. This wide-ranging approach, while comprehensive, may dilute the specificity and depth of analysis that could be achieved by focusing on a singular application. In future studies, narrowing the scope to concentrate on one specific application might yield more conclusive and detailed results.

Research related to certain applications, such as laser-assisted ovate pontic site preparation or the use of photobiomodulation (PBM) therapy in prosthodontics with fixed partial dentures, remains limited. Furthermore, the majority of the evidence supporting these applications primarily comes from case studies. While valuable, they generally provide less robust evidence compared to controlled trials, necessitating more reliable and rigorous research methods to substantiate their findings.

Additionally, certain applications are currently only supported by in vitro studies using extracted teeth. The relevance of these studies is constrained, as the response of a living tooth to laser technology may vary significantly from that observed in extracted teeth. More in vivo research is needed to assess the true efficacy and safety of these technologies.

Establishing a definitive protocol across all applications remains a challenge due to significant variations in the applied parameters, including the duration, session intervals, modes, and types of lasers used in the available studies. Research authors should be expected to provide more rigorous data presentation, enabling both the replicability and applicability of their studies. This enhanced clarity and detail in documenting findings is crucial for advancing scientific understanding and practical application.

Narrative reviews, by their nature, are limited in terms of objectivity, the completeness of the literature search, and the interpretation of findings. Unlike systematic reviews, which utilize explicit methods to search and evaluate the literature, narrative reviews may not fully capture all relevant studies, potentially leading to biases in the conclusions. The current study attempts to present information with as much objectivity and clarity as possible; however, the inherent limitations of the narrative review format mean that the findings should be interpreted with caution. Future research could address these limitations by employing more rigorous methodologies that enhance reproducibility and reduce biases.

## 4. Conclusions

Lasers can be applied at many stages of prosthodontic rehabilitation with fixed dental restorations. They demonstrate excellent and desirable clinical properties in dentistry, such as low invasiveness, biocompatibility, and high precision. Due to these features and the ability to manipulate both soft and hard tissues, there is a range of procedures which benefit from the intraoral usage of lasers. This technology allows dentists to conduct treatments with great precision, high efficiency, and meticulous care, contributing to the permanent gingival remodeling, healthy periodontium, and durable restorations. While lasers offer a compelling alternative to multiple conventional methods, certain procedures, such as the damage-free removal of ceramic crowns or gum photobiomodulation, can only be executed using laser technology. They are well tolerated by patients, ensure comfort during the procedure, and provide satisfactory esthetic results. A complete summary of laser applications in dental prosthodontics is illustrated in the Table 4.

Although lasers offer numerous advantages, they also come with several drawbacks. The high cost of purchasing and maintaining this equipment directly contributes to the expensive nature of treatments for patients. Additionally, operating lasers requires specialized knowledge and precision, necessitating strict adherence to safety protocols to mitigate any harmful effects from laser emissions. Both clinicians and patients must wear protective laser glasses to safeguard their eyesight during procedures. This requirement for extra precautions means that additional staff training is necessary, further increasing costs. Despite their prevalence, lasers have limitations that prevent them from fully replacing conventional tools in dental practices.

## Figures and Tables

**Table 1:** Parameters of dental lasers.

Laser Type	CO_2_	Er:YAG	Er,Cr:YSGG	Nd:YAG	Argon	Diode	Helium Neon
Wavelength	10,600 nm	2940 nm	2780 nm	1064 nm	488–514 nm	655–980 nm	637 nm
Mode	Pulse or continuous-wave	Pulse	Pulse	Pulse	Pulse or continuous-wave	Pulse or continuous-wave	Continuous-wave
Spectrum	Infrared	Infrared	Infrared	Infrared	Blue–blue/green	Red–Infrared	Red
Laser active material	Solid	Solid	Solid	Solid	Gas	Semiconductor	Gas
Level of energy emission	High	High	High	High	High	High and low	Low

**Table 2:** Presentation of benefits and drawbacks of dental lasers.

Laser Type	Advantages	Disadvantages
CO_2_	Exhibits very high water absorption, enabling quick removal of soft tissue and effective hemostasis with minimal depth penetration Achieves the highest absorption in hydroxyapatite Rarely causes bleeding Delivers excellent beam quality Effectively controls hemorrhage	High cost
Erbium laser	No anesthesia required Painless for the patient Features the highest water absorption among lasers High affinity for hydroxyapatite Excellent beam quality Wide range of emission power Suitable for soft tissues with its high water absorption capability	Limited hemostatic ability
Nd:YAG	Excellent beam quality Broad spectrum of emission power Safely operates near healthy tooth structures without causing damage Operable with or without direct contact Effective in achieving good hemostasis	Exhibits low absorption in water Inferior at cutting hard tissues compared to erbium lasers Emitted light is minimally absorbed by amalgam, titanium, and other non-precious metals Best used for slower production processes with thicker materials, which results in lower efficiency
Argon	Operates at two emission wavelengths: 488 nm and 514 nm Ideal for less invasive soft tissue procedures Offers effective hemostasis	Laser light delivery crosses tissue barriers, irradiating tissues beyond the intended target
Diode	Versatile with a broad spectrum of wavelengths Operable in both pulse- and continuous-wave modes Exhibits greater water absorption in dental tissues compared to Nd:YAG lasers Achieves hemostasis more slowly than Argon lasers Features poor absorption by tooth structures, facilitating soft tissue surgery near enamel, dentine, and cementum	Poor beam quality

**Table 3:** Parameters of three lasers used for gingival troughing by Tao et al. [41].

Laser	Wavelength	Mode	Power (W)	Frequency	Tip (μm)	Cool
Diode	810	Continuous pulse	2	20	320	No
Nd:YAG	1064	Short pulse	2	15	320	No
Er:YAG	2940	Very long pulse	2	15	500	Air and water

**Table 4:** Overview of lasers along with their use in fixed prosthodontics. A plus sign (+) in the table indicates a possible application in the procedure.

	Prosthodontic Applications								
Laser Type	Gingivectomy	Preparation of Ovate Pontic Site	Teeth Whitening	Gingival Troughing	Surface Conditioning	Dentine Hypersensitivity Treatment	PBM and Tissue Regeneration	Removal of Ceramic Dental Restorations	Surface Decontamination
Diode laser	+	+	+	+		+	+		
Er:YAG	+			+	+	+		+	+
CO₂	+		+		+	+			
Er,Cr:YSGG	+				+			+	+
Argon laser			+						
Nd:YAG				+	+	+			+
He-Ne						+			

## Data Availability

No new data were created or analyzed in this study. Data sharing is not applicable to this article.
